# Building Asymmetric Lipid Bilayers for Molecular Dynamics Simulations: What Methods Exist and How to Choose One?

**DOI:** 10.3390/membranes13070629

**Published:** 2023-06-29

**Authors:** Emily H. Chaisson, Frederick A. Heberle, Milka Doktorova

**Affiliations:** 1 Department of Chemistry, University of Tennessee Knoxville, Knoxville, TN 37916, USA; 2 Department of Molecular Physiology and Biological Physics, University of Virginia, Charlottesville, VA 22903, USA

**Keywords:** membrane asymmetry, differential stress, interleaflet coupling

## Abstract

The compositional asymmetry of biological membranes has attracted significant attention over the last decade. Harboring more differences from symmetric membranes than previously appreciated, asymmetric bilayers have proven quite challenging to study with familiar concepts and techniques, leaving many unanswered questions about the reach of the asymmetry effects. One particular area of active research is the computational investigation of composition- and number-asymmetric lipid bilayers with molecular dynamics (MD) simulations. Offering a high level of detail into the organization and properties of the simulated systems, MD has emerged as an indispensable tool in the study of membrane asymmetry. However, the realization that results depend heavily on the protocol used for constructing the asymmetric bilayer models has sparked an ongoing debate about how to choose the most appropriate approach. Here we discuss the underlying source of the discrepant results and review the existing methods for creating asymmetric bilayers for MD simulations. Considering the available data, we argue that each method is well suited for specific applications and hence there is no single best approach. Instead, the choice of a construction protocol—and consequently, its perceived accuracy—must be based primarily on the scientific question that the simulations are designed to address.

## 1. Introduction

Lipid bilayers are ubiquitous in biology. Constituting the core of cellular membranes, they have long fascinated scientists trying to uncover their multifaceted roles in biological processes. Of particular interest are membrane biophysical properties and their relation to phenomena such as protein-lipid interactions and selective solute permeability [1,2]. In this context, the effects of lipid composition—one of the major determinants of the bilayer’s structural and mechanical properties—have been extensively studied in symmetric model membranes (defined as having two leaflets with identical lipid composition and number density) that can be easily prepared and manipulated [3].

**Figure 1:**
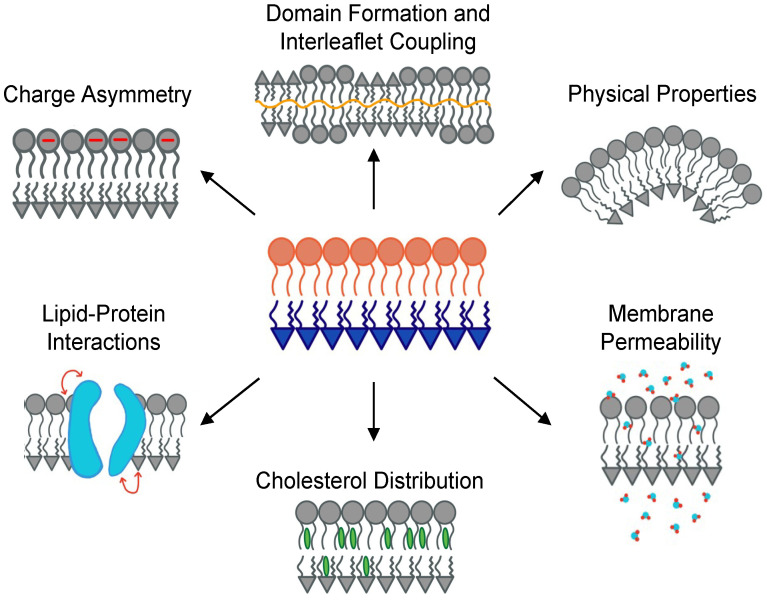
Various properties of asymmetric membranes studied with experiments and simulations. Clockwise from top: Effects of asymmetry on phase separation and interleaflet communication and adjustments; physical properties such as curvature, rigidity, and differential stress; permeability of water and small molecules; distribution of cholesterol between the two leaflets; interaction of membrane proteins with the bilayer; and charge asymmetry.

A major discovery in the early 1970s found that the plasma membrane of erythrocytes is compositionally asymmetric, with sphingomyelin and phosphocholine lipids enriched in the outer leaflet, and the aminophospholipids PS and PE largely confined to the inner leaflet [4,5]. These observations were later confirmed for the plasma membranes of many eukaryotic cells [6], which necessitated the development of novel experimental and theoretical paradigms for the investigation of membrane asymmetry [7,8,9,10]. Central questions in early studies concerned the effects of asymmetry on protein-membrane interactions and interleaflet coupling of phase behavior (Figure 1) [11,12,13,14,15,16,17]. 

Offering an atomic resolution that is difficult to achieve with experiments, molecular dynamics (MD) simulations have often provided invaluable insights into the origins of membrane behavior and properties (e.g., [18,19,20,21,22,23]). Therefore, it is not surprising that the computational investigation of membrane asymmetry quickly gained speed alongside the rather challenging experimental characterization of asymmetric model membranes. Inspired mainly by observations in cells, some of the first MD simulation studies focused on the effects of asymmetry on membrane electrostatics and permeability [24,25,26,27], the structural properties of asymmetrically distributed gangliosides [27], and the peculiar confinement of raft-forming mixtures to the outer plasma membrane leaflet [28]. Later, advances in related experimental techniques [7] stimulated the investigation of interleaflet coupling [10,29,30,31,32,33,34,35,36,37,38], cholesterol interleaflet distribution [36,39,40,41,42,43], and protein interactions with asymmetric bilayers [44,45,46,47,48,49,50] (Figure 1).

In an MD simulation, a molecular model is first constructed, and its dynamics are simulated by evaluating inter-atomic forces from sets of parameters (often experimentally calibrated) and propagating the system in time following Newton’s laws of motion. Freely available software packages have been developed to assist with both steps, making MD simulations and their analysis readily accessible [51,52,53,54,55,56,57,58,59,60]. Although an asymmetric bilayer with well-defined leaflet lipid compositions can be built in minutes and generally simulated without major hurdles, it has become clear that one decision in the initial bilayer design—namely, the relative numbers of slowly-flipping lipids to place in the two leaflets—can be more consequential even than the choice of the specific lipid compositions of the leaflets [10]. Below, we describe this challenge and review various protocols that have been devised for constructing asymmetric lipid bilayers for MD simulations. We then outline a strategy for choosing the most appropriate construction method in the pursuit of simulation studies of membrane asymmetry.

## 2. The Main Challenge

Due to their amphiphilic nature, lipid membranes exist in a closed geometry—that is, the two ends of a membrane sheet meet to form a continuous surface such as those encapsulating a liposome or a cell. While large-scale simulations of vesicles can be performed given ample computational resources [61,62], simulation studies of lipid bilayers are traditionally performed with relatively small bilayer patches having 50–200 lipids per leaflet. This is achieved by using periodic boundary conditions (PBC), where a unit cell containing the molecular system is surrounded by periodic images so that a particle that exits from one side of the unit cell immediately enters it again from another (often the opposite) side. This mathematical representation eliminates interfaces and helps mimic an infinite membrane sheet out of a small bilayer patch, thus removing any edge effects imposed by the finite system size.

**Figure 2:**
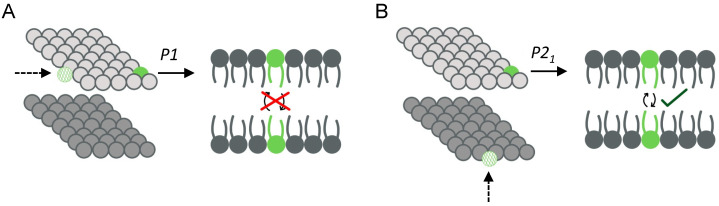
Different periodic boundary conditions (PBCs) used in bilayer simulations. (**A**) Standard P1 PBCs commonly used in MD simulations of lipid bilayers. When a lipid exits the unit box from one side it reappears on the opposite side in the same leaflet, prohibiting free exchange of lipids between leaflets. (**B**) Modified P2_1_ PBCs where the primary simulation cell is rotated and translated to allow free passage of lipids between leaflets. When a lipid exits the unit box from one side it reappears in the opposite leaflet via an orthogonal face allowing sampling of both leaflet environments. Schematics on the left in (**A**,**B**) were adapted with permission from Ref. [63] (2002, Elsevier) and illustrate the lipid headgroups on the two leaflets.

The standard PBCs used in lipid bilayer simulations are referred to as P1 boundary conditions and are depicted in Figure 2A [63]. This treatment approximates an infinite membrane where lipids diffuse freely within the lateral plane of their respective leaflets. Transverse lipid diffusion (i.e., flip-flop) can occur spontaneously but is typically a rare event on the current timescales of atomistic simulations, consistent with experimental studies that report slow flip-flop rates [64] (cholesterol is a notable exception discussed below). In contrast, a different type of PBCs referred to as P2_1_ boundary conditions, allows lipids to readily exchange between leaflets as when they exit the unit cell from one side, they reappear not only on a different side but also on the opposite leaflet (Figure 2B). The P2_1_ PBCs are currently implemented only in the CHARMM software [65] and allow for a specialized construction and simulation of asymmetric bilayers as discussed below.

To allow the bilayer to ‘breathe’ and stay relaxed (i.e., maintain a net surface tension of zero) in the presence of thermal energy, simulations are usually performed in the NPT ensemble of constant pressure and temperature with semi-isotropic pressure coupling. That is, the pressure in z (the direction normal to the bilayer plane) is kept constant, while the pressures in x and y are identical and change simultaneously. This leads to the continuous expansion and compression of the bilayer area with the respective fluctuations governed by the bilayer area compressibility modulus [66].

Due to the applied PBCs, the area fluctuations of the two leaflets are constrained to be the same as in the membrane of a liposome, for example [22,67]. However, in contrast to liposomes whose membrane can undergo large morphological changes in response to mismatched leaflet areas [68,69,70,71], the responses of a relatively small bilayer patch are more limited. Thus, if leaflet A in a simulated bilayer has fewer lipids, the chains of those lipids will splay out to decrease the effective lipid packing density so that the total leaflet area matches that of the opposing leaflet B. Similarly, the lipids in leaflet B will reduce the cross-sectional area of their chains (i.e., compress) to facilitate the mutual adjustment. This was clearly illustrated in studies that investigated the stability of simulated bilayers upon systematic removal of lipids from one leaflet [49,72]. Even in the presence of a large lipid number mismatch, the leaflets adjusted their packing densities accordingly and the bilayer maintained its integrity.

Apart from the complementary lipid packing adjustments of the leaflets, what is notably different in these mismatched simulations is the accumulation of tension in both leaflets as a result of the suboptimal lipid areas. Leaflet A with fewer, splayed lipids develops positive tension $\;(\tau_{A}>0$), while leaflet B with compressed lipids develops negative tension or pressure ($\tau_{B}<0$). Since the pressure in z is constant and no external forces are applied to the membrane, the tensions in the two leaflets have the same magnitude but opposite sign $\;(\tau_{A}=-\tau_{B}$) such that $\tau_{A}+\tau_{B}=0$. In a symmetric bilayer with the same composition and lipid number density in each leaflet, $\tau_{A}=\tau_{B}=0$. The non-zero leaflet tension in bilayers with either number or compositional asymmetry (or both) has been termed *differential stress* [10]. It is associated with differential changes (expansion and compression) in the packing, as well as overall properties, of the two leaflets as illustrated by the single-component membranes with mismatched numbers of lipids [49,72]. While both differential stress and any potential curvature stress in the membrane (i.e., arising from differences in the spontaneous curvatures of the two leaflets) may be alleviated if the bilayer adopts a non-zero curvature, the applied periodic boundary conditions can effectively suppress such bending and result in the accumulation of additional compensating differential stress [10].

While the presence and effects of differential stress can be clearly seen in the compositionally symmetric systems just described, detecting differential stress in compositionally asymmetric bilayers is non-trivial. In that case, depending on the respective leaflet lipid compositions, equal numbers of lipids in the two leaflets can lead to significant differential stress that can be alleviated by introducing a particular number mismatch, resulting in a bilayer with zero leaflet tension [35,67,73,74]. This is due to the inherent variability in the structure and properties of different lipid types, as well as to the effects of interleaflet coupling which may change those properties in ways that are not yet fully understood. Molecules such as cholesterol are also capable of reducing the stress in the bilayer, however, they may not always do so as we discuss below. Importantly, due to the prohibitively slow exchange of phospholipids between leaflets in atomistic simulations with the standard P1 boundary conditions, the lipids retain their leaflet residence for the entirety of the simulation (one notable exception is the use of the P2_1_ boundary conditions shown in Figure 2B and discussed below). While this is precisely what enables the simulation of asymmetric bilayers of defined lipid compositions, it also makes the initial bilayer construction a critical determinant of the presence and related effects of differential stress. In other words, the properties of a simulated asymmetric bilayer depend not only on its composition but also (and to a large extent) on how it is constructed.

## 3. Protocols for Bilayer Construction

To date, there are four main approaches for the construction of compositionally asymmetric bilayers for MD simulations:

1. Ensure equal numbers of lipids in the two leaflets (**EqN**).
2. Match the surface areas (or lipid packing densities) of the two leaflets to those from cognate symmetric bilayers (**SA**).
3. Eliminate differential stress, i.e., ensure zero leaflet tension (**0-DS**).
4. Emulate biological asymmetry (**EmBioAsym**).

Most of these methods require performing at least one simulation to arrive at the desired asymmetric membrane model. Once the relative leaflet compositions and abundances are determined, the bilayer can be simulated with any conventional software to investigate its properties in detail. Below we briefly describe each of these approaches and discuss a few studies in which they have been applied.

### 3.1. Same Number of Lipids (EqN)

**Figure 3:**
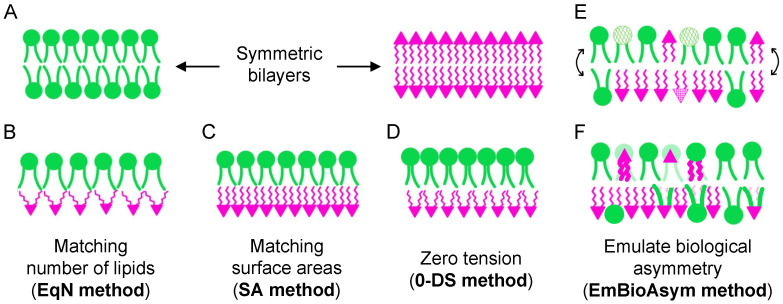
Protocols for the construction of asymmetric bilayers for MD simulations. (**A**) Symmetric bilayers have the same number and type of lipids in their two leaflets. An asymmetric bilayer can be built (**B**) with equal numbers of lipids in the two leaflets (**EqN** method) or (**C**) by ensuring that the relative packing densities (or surface areas) of the two leaflets are initially the same as in cognate symmetric bilayers (the **SA** method). Alternatively, (**D**) iterative simulations and analysis can be used to identify the leaflet number asymmetry that eliminates differential stress (the **0-DS** method) or (**E**,**F**) advanced simulations can be performed to optimize the leaflet lipid compositions. The latter is achieved by letting a subpopulation of the lipids equilibrate their leaflet concentrations by either (**E**) freely exchanging between leaflets or (**F**) changing their identity, in the presence of constraints keeping other lipids in place (e.g., lipids with patterned headgroups in (**E**) can diffuse only within their respective leaflets).

Since in symmetric bilayer simulations, the two leaflets generally have equal numbers of lipids (Figure 3A), one approach to building asymmetric bilayers is to ensure that same condition (Figure 3B). This can be accomplished by building a symmetric bilayer and replacing individual lipids with different ones, or by specifying the same total numbers of lipids in the two leaflets and letting software generate the asymmetric lipid compositions. Here, all lipids are treated identically, thus disregarding any structural differences or packing preferences between species. This approach has been used to study the physical properties of both simpler lipid mixtures [27,30] and asymmetric plasma membrane models of increasing complexity [75,76].

### 3.2. Match Surface Areas (SA)

An alternative to the **EqN** method is choosing the relative numbers of lipids in the two leaflets so that at the initial stage of bilayer construction, their average areas per lipid (or surface areas) match those of cognate symmetric bilayers (Figure 3C). This can be accomplished by first simulating two symmetric bilayers with the lipid compositions of the asymmetric membrane leaflets, followed by either stitching their leaflets together or using the obtained equilibrium areas per lipid to calculate the respective numbers of lipids and building the asymmetric bilayer from scratch. The latter approach is more general and allows for the construction of asymmetric bilayers of different sizes. This method ensures that the relative packing densities of the two leaflets remain fixed even as the two individual leaflet areas may simultaneously increase or decrease in comparison to their symmetric counterparts. This protocol has been applied to the analysis of the membrane potential [24,25,26], interleaflet coupling [29,32,34], the permeability of plasma membrane models [77], and cholesterol interleaflet distribution [39,42].

To avoid simulating two symmetric bilayers, one can alternatively use reported areas per lipid (APL) for the individual lipids and, if the leaflets contain more than one component, assume ideal mixing and calculate the respective mole-fraction-weighted packing densities. For instance, CHARMM-GUI [54] uses individual lipid APLs to estimate the number of lipids in each leaflet when only lipid composition and lateral box dimensions are provided. This approach was recently used in a study comparing different construction methods for asymmetric bilayers [8].

### 3.3. Eliminate Differential Stress (0-DS)

Recent experiments have found that preferred lipid packing densities in symmetric bilayers can be altered in asymmetric bilayers, presumably due to the effects of the interleaflet coupling [78]. A corollary is that lipid areas in asymmetric bilayers cannot be estimated a priori from their values in symmetric bilayers. It follows that the **EqN** and **SA** methods described above may produce bilayers with differentially stressed leaflets [67,73]. To ensure zero leaflet tension (Figure 3C), one can follow the protocol outlined in [67], which is based on the following principle. An asymmetric bilayer is first constructed (for example, using the **EqN** or **SA** methods) and simulated until the APL is converged over the last ~200 ns. Then, the lateral pressure profile is calculated from the converged portion and used to obtain the leaflet tension. If the tension is non-zero, the number of lipids is adjusted by either removing lipids from the leaflet with negative tension or adding lipids to the leaflet with positive tension. The exact number of lipids to add or remove can be chosen on a trial-and-error basis or estimated from the relationship between the bilayer area compressibility modulus and leaflet tension (Equation 3 in [67]). A new asymmetric bilayer is then built from scratch with the updated leaflet lipid numbers and the same steps are repeated (i.e., simulation, calculation of leaflet tension, adjustment, and rebuilding if necessary) until zero leaflet tension is reached. This approach has been used to examine the effects of asymmetry on the mechanical properties of anionic asymmetric bilayers [74] and the ability of gramicidin to scramble lipids [46], as well as the effects of differential stress on the interaction of small molecules with asymmetric membranes [73].

### 3.4. Emulate Biological Asymmetry (EmBioAsym)

The asymmetry in cell plasma membranes (PM) is maintained via the activity of flippase and floppase enzymes which, when active, move lipids between leaflets against their concentration gradients [79]. Since the lipid specificity of these enzymes is arguably restricted to certain lipid types [80], one hypothesis is that cells regulate their PM lipid organization by restricting the asymmetry of some lipids while letting others equilibrate between leaflets according to their chemical potential. One notable example is cholesterol, a major component of mammalian cell plasma membranes that can rapidly flip between leaflets [6,81,82,83]. Interestingly, while cholesterol is capable of alleviating stresses in the membrane [84], its strong preference for interaction with saturated lipids may dominate over elastic and entropic forces and drive its distribution in a way that *increases* the differential stress [10,36,85]. This illustrates both the natural tendency of a bilayer constituent to equilibrate its distribution based on its chemical potential and the fact that realizing this tendency may produce rather than eliminate stresses in the membrane. In that respect, two methods have emerged to examine the equilibration of lipid redistribution in a simulated bilayer in the presence of imposed asymmetry [8,9].

The first approach involves simulations in the NPT ensemble and utilizes P2_1_ boundary conditions (Figure 3E) [8]. These PBCs allow lipids to sample both leaflet environments by freely exchanging between leaflets during the simulation (Figure 2B) [63]. To mimic the activity of flippases and simulate asymmetric membranes, the method involves constraining some lipids to stay in one leaflet while allowing others to equilibrate their leaflet concentrations via an interleaflet redistribution [8]. Thus, it is possible to start with a bilayer constructed with one of the methods described above, then restrict some lipids to their respective leaflets and simulate the system with P2_1_ PBCs to examine the preferred lipid distribution of the unconstrained membrane components in the presence of the imposed asymmetry. Consequently, since the relative numbers of lipids in the two leaflets are not constrained, they can dynamically change during the simulation. As noted by the authors, while opening transient pores in the membrane can also accelerate the exchange of lipids between leaflets, the advantage of P2_1_ PBCs is that the chemical equilibrium reached by the freely diffusing lipids is a property of the asymmetric leaflets in the absence of any mechanical perturbations such as those imposed by a pore [8].

The second approach starts with a compositionally symmetric bilayer and replaces some of the lipids with new ones to generate the initial asymmetry (if the bilayer contains the same lipid numbers across leaflets, this is equivalent to the **EqN** method). It then proceeds with a simulation not in the NPT ensemble (as discussed above), but instead in a *semi-grand canonical* ensemble that allows dynamic changes in lipid identity (specifically their saturation or headgroup type) during the simulation (Figure 3F) [9]. This approach emulates the action of lipid-translocating enzymes by imposing a chemical potential difference between some molecules in one leaflet while letting the leaflet lipid compositions adjust in accordance with the chemical potential of all lipid species subject to the imposed constraints. In these simulations, the lipid number asymmetry is fixed, but the changes in the degree of lipid saturation or type across species dynamically alter the relative packing densities of the leaflets by virtue of their changing lipid compositions. This method is well suited for investigating how some asymmetries might naturally arise from others and has thus helped explain certain experimental observations of the leaflet lipid compositions in erythrocyte membranes [9].

## 4. What Is the Best Approach?

Considering the very different approaches for constructing and equilibrating asymmetric bilayers discussed above, it is natural to question whether one method is superior to the others. The **EqN** method is the simplest to implement, whereas the **SA** method relies on either prior knowledge of lipid areas or short simulations of symmetric membranes to obtain areas (though freely available software packages can tackle this demand easily). The **0-DS** method is more involved as it requires a few iterations of simulation and analysis, while the implementation of the **EmBioAsym** approach is currently possible only on specific software platforms (the HOOMD-Blue molecular dynamics engine [86] for simulations in the semi-grand canonical ensemble and CHARMM [65] for applying P2_1_ boundary conditions) and requires advanced simulation techniques (Table 1). Although **EqN** is a clear winner from the standpoint of simplicity, we now argue that there is no single best approach. Instead, the choice of how to construct an asymmetric bilayer should depend primarily on the scientific question motivating the simulation, as illustrated by the examples below.

The nature of interleaflet coupling and the effects of asymmetry on membrane properties have been the focus of many model membrane studies. The three cognate symmetric bilayers (i.e., representing the compositions of each leaflet separately as well as the overall average bilayer composition) constitute a baseline for understanding how leaflet properties change when compositional asymmetry is imposed. Since symmetric bilayers by definition have zero leaflet tension, and different amounts of differential stress have distinct effects on the properties of an asymmetric bilayer [35,73], this comparison is most informative when carried out with stress-free asymmetric membranes [67]. As such, the **0-DS** method is the most appropriate as it guarantees the absence of differential stress and thus isolates the effects of asymmetry. Moreover, if the goal is to study a bilayer with specific fixed leaflet compositions (for example, to compare with results from model membrane experiments where leaflet lipid compositions have been quantified), the **EmBioAsym** methods will not be practical since leaflet compositions change during the course of the simulation.

Motivated by the asymmetry observed in biological membranes, other studies have focused on understanding the biophysics of more complex asymmetric membranes [75,76,87]. Here, the point of reference is usually the biological membrane of interest, for example, the plasma membrane (PM) of eukaryotic cells. Importantly, there are currently no experimental data for the extent of differential stress in the PM or any other biological membrane, and ensuring zero leaflet tension in these studies is, therefore, not yet warranted. Moreover, the relative numbers of lipids in the two PM leaflets may be far from equal [36,85], and the interleaflet distribution of cholesterol has not been conclusively established for the PM [82]. As a result, the extent to which the lipid packing densities of the asymmetric PM leaflets deviate from their values in cognate symmetric bilayers is unclear. It is reasonable to conclude that neither the **EqN**, **SA**, or **0-DS** methods are justified when constructing a PM model. At the same time, it is well established that cells actively maintain the asymmetric distribution of certain lipid classes [6,88]. Emulating the activity of lipid-translocating enzymes and letting the non-regulated membrane components equilibrate their concentrations according to their chemical potential would therefore produce the most relevant model of a biological membrane.

The considerations just outlined suggest that the **EmBioAsym** approaches may be best suited for studying questions specifically pertaining to complex biological membranes. While each of the **EmBioAsym** methods requires a simulation (one in the semi-grand canonical ensemble, and one with P2_1_ boundary conditions) with constraints to arrive at the desired membrane state, they differ in the nature of their assumptions and the questions they can potentially answer. For example, the method of imposing a chemical potential difference between lipids in a leaflet (Figure 3F) [9] has helped explain the surprising increase in chain unsaturation of PC lipids in the exoplasmic PM leaflet of red blood cells as well as the tendency of plasmalogen lipids to be highly unsaturated [88]. In these simulations, the phospholipid number asymmetry in the two leaflets remains fixed, but the effects of any number imbalances can be modeled by performing multiple simulations with different starting configurations. The resulting changes in lipid chain saturation and headgroup type (i.e., the optimized leaflet lipid compositions) are tightly coupled to the magnitude of the imposed chemical potential difference and the two reference lipid classes that define it, allowing for systematic studies of the effects of different constraints. Limitations of this approach include the relatively narrow range of phospholipid number asymmetries that can be modeled due to bilayer stability issues (e.g., [71]) and the computational demands of the simulations which currently restrict them to a coarse-grained representation.

In contrast, P2_1_ boundary conditions (Figure 2B) that allow lipids to freely move between leaflets are implemented in CHARMM with all-atom membrane models. Simulations are not as computationally costly, are performed in the standard NPT ensemble, and allow for the numbers of lipids in the two leaflets to change dynamically, thus obviating the need to impose a certain phospholipid number asymmetry a priori (Figure 3E). Since the simulation setup allows lipids to sample the two leaflets and equilibrate their distribution, the final energy-minimized bilayer model is dependent on the overall bilayer composition and can technically be reached starting from different initial leaflet asymmetries. Importantly, a subpopulation of lipids needs to be confined to one leaflet, and thus the choices of which lipids to constrain and how to constrain them will influence the final result. In a recent study that utilized P2_1_ boundary conditions, the authors applied two flat-bottom potentials to a subset of lipids, effectively letting them move freely within their respective leaflets but pushing them back when they approached the edge of the simulation box [8]. This constraint precludes the calculation of some dynamical properties such as lipid diffusion. However, similar to the approach that utilizes a semi-grand canonical ensemble, once the compositions of the two leaflets have converged in the constrained simulations, the asymmetric bilayer can be simulated constraint-free with the standard P1 PBCs (Figure 2A) for calculation of any properties of interest.

The **EqN** and **SA** methods can also be used to generate asymmetric bilayers and study their properties. Though they require little a priori information, the questions they can address are more limited. Perhaps most importantly, the simulated membranes may harbor differential stress due to the fixed number of lipids in their leaflets. Still, the results can be generalized to other findings if placed in the context of this differential stress. For example, if the leaflets do not change their relative packing densities in an asymmetric bilayer, how stressed would they be based on their lipid compositions? Or, if the exchange of lipids in the outer leaflet of vesicles catalyzed with e.g., cyclodextrin is 1:1 (that is, the two leaflets of the asymmetric liposomes retain equal numbers of lipids), what would be the resulting differential stress in the membrane? A clear definition of the purpose of the simulation and awareness of the advantages and disadvantages of all available methods for bilayer construction can thus help identify the most optimal approach (Table 1).

## 5. Ease of Implementation and Future Challenges

The existing protocols for constructing and simulating asymmetric membranes described above can be used to address a wide range of scientific questions (Table 1). All except for the **EmBioAsym** approaches are readily accessible with conventional software packages such as CHARMM-GUI for the initial construction of the models and NAMD [55], GROMACS [57], and OpenMM [60] for their simulation and analysis. Further technical developments would be needed to implement the **EmBioAsym** approaches in these and other molecular dynamics engines and make them more generally accessible to non-experts. Even then, however, there are still some outstanding challenges facing all existing methodologies. For example, the treatment of lateral heterogeneities—both in terms of the formation and coexistence of domains in one or both leaflets—presents many unknowns concerning lipid packing, interleaflet coupling, differential stress, and domain stability, thus limiting the direct applicability of existing approaches (see e.g., [29]). The effects of asymmetry on the membrane curvature and overall membrane morphology represent yet another mostly unexplored aspect of asymmetry that may necessitate the refinement of existing methods or the development of new ones. Construction and simulation of sufficiently large asymmetric bilayers, including liposomes, that allow for large-scale morphological changes while controlling for differential stress or emulating biological asymmetry as in flat membrane patches constitute the next big step towards simulations of more realistic asymmetric membranes [89]. The tools and approaches available today provide a springboard for gathering the information needed to address these big challenges in the years to come.

## 6. Conclusions

The protocol used to construct an asymmetric bilayer in silico has a significant impact on the resulting bilayer properties and the conclusions that can be drawn from them. This is due to both technical issues (e.g., applied periodic boundary conditions, limited timescales) and physical-chemical factors inherent to lipid bilayers (e.g., slow spontaneous lipid flip-flop). Different types of methods exist to aid in this task, but which method is most appropriate depends strongly on the scientific intent of the simulations. Each approach has advantages and disadvantages, and each is uniquely suited to address specific types of questions (Table 1). Notably, the asymmetric bilayers generated with these methods are not expected to be the same since they rely on different assumptions and indirect or direct constraints to ensure asymmetric lipid distribution. However, the advantage of any model membrane study is that parameters can be clearly defined and systematically varied, and performing multiple simulations can in principle address the limitations of a given model. For example, if experimental data exist for validation, one can vary the numbers of lipids in one leaflet for the methods that maintain a constant lipid number asymmetry or constrain different groups of lipids in the **EmBioAsym** approaches, to find the best match with experimentally determined properties.

Unlike symmetric bilayers, asymmetric bilayers are not completely defined by their lipidome [10]. Instead, two asymmetric bilayers with the same leaflet lipid compositions can have very different properties depending on the amount of differential stress [67,73]. Therefore, both the leaflet lipid compositions and the relative numbers of lipids in the two leaflets (or equivalently, the resulting differential stress) are needed to fully characterize an asymmetric membrane. Controlling for, or at least reporting, the differential stress of an asymmetric membrane—be it in silico, in vitro, or in vivo—is necessary for placing the results in the right context. Although this is currently possible only in simulated systems, recent advances raise the possibility of experimental estimates of differential stress in the not-too-distant future [90]. Importantly, even though the differential stress in biological membranes is presently unknown, much can still be learned from simulations of asymmetric model membranes if the method for constructing these models is chosen appropriately.

## Figures and Tables

**Table 1:** Summary of the advantages, disadvantages, and suitable applications of the different methods discussed in the text. Note that in the **EqN**, **SA**, and **0-DS** methods, the asymmetric distribution of all lipids remains fixed throughout the simulation with the exception of cholesterol, which can efficiently redistribute and sample both leaflets.

Method	Pros	Cons	Suitable Applications
**EqN**	Does not require prior information or simulations	Does not consider differences in lipid packing preferences May produce membranes with varying amounts of differential stress	Biophysical properties of asymmetric model membranes prepared from symmetric membranes via 1-to-1 exchange of outer leaflet lipids with new ones
**SA**	Accounts for relative leaflet packing preferences from symmetric membranes	Requires prior simulations or information about lipid packing densities May produce membranes with varying amounts of differential stress	Biophysical properties of asymmetric model membranes in which the relative packing densities of the two leaflets match those from their respective symmetric counterparts
**0-DS**	Produces membranes with no differential stress	May require multiple simulations and analysis	Effects of asymmetry on bilayer biophysical properties based on comparisons between asymmetric and symmetric membranes
**EmBioAsym**	*P2_1_ PBC*		
	Allows lipids to sample both leaflets and equilibrate their respective leaflet concentrations	Requires a simulation with P2_1_ PBCs currently possible only in CHARMM Requires constraints on the asymmetry of some lipids	Lipid distribution and biophysical properties of asymmetric membranes with fixed overall lipid composition in which the asymmetric distribution of some lipids is actively maintained (i.e., stays constant)
	*Semi-grand canonical ensemble*		
	Allows leaflet lipid compositions to change dynamically and equilibrate according to chemical potentials	Requires a simulation in the semi-grand canonical ensemble currently possible only with coarse-grained models in HOOMD-blue Requires constraints on the chemical potential difference of some lipids	Leaflet lipid compositions and biophysical properties of asymmetric membranes in which the asymmetric distribution of some lipids is actively maintained (i.e., stays constant) while the overall lipid composition can change due to e.g., the activity of lipid-modifying enzymes or access to extra lipid pools

## Data Availability

Data sharing not applicable.
